# Catastrophic out-of-pocket payments for dental treatment: regional evidence from Spain

**DOI:** 10.1186/s12913-023-09761-5

**Published:** 2023-07-22

**Authors:** Samuel López-López, Raúl del Pozo-Rubio, Marta Ortega-Ortega, Francisco Escribano-Sotos

**Affiliations:** 1 Castilla-La Mancha Health Services, SESCAM, Cuenca Hospital. C/ Hermandad de Donantes de Sangre, 1. 16.002, Cuenca, Spain; 2 Department of Economic Analysis and Finance, University of Castilla-La Mancha, Avda, Los Alfares, 44, 16.071, Cuenca, Spain; 3 Research Group On Food, Economy and Society, University of Castilla-La Mancha, Cuenca, Spain; 4 Department of Applied and Public Economics and Political Economy, Complutense University of Madrid, Campus de Somosaguas S/N. 28.223, Pozuelo de Alarcón, Madrid, Spain; 5 Department of Economic Analysis and Finance, University of Castilla-La Mancha, Plaza de La Universidad S/N. 02.001, Albacete, Spain

**Keywords:** Catastrophic, Out-of-pocket payments, Dental treatment, Public health service, Region, I11, I18, I32

## Abstract

**Background:**

To estimate the incidence and concentration of catastrophic out-of-pocket payments for healthcare and dental treatment, by region in Spain (calculated as the proportion of households needing to exceed a given threshold of their income to make these payments) in 2008, 2011 and 2015.

**Methods:**

The data analysed were obtained from the Spanish Family Budget Survey reports for the years in question. The study method was that proposed by Wagstaff and van Doorslaer (2003), contrasting payments for dental treatment versus household income and considering thresholds of 10%, 20%, 30% and 40%, thus obtaining incidence rates. In addition, relevant sociodemographic variables were obtained for each household included in the study.

**Results:**

With some regional heterogeneity, on average 4.75% of Spanish households spend more than 10% of their income on dental treatment, and 1.23% spend more than 40%. Thus, 38.67% of catastrophic out-of-pocket payments for dental services in Spain corresponds to payments at the 10% threshold. This value rises to 55.98% for a threshold of 40%.

**Conclusions:**

An important proportion of catastrophic out-of-pocket payments for health care in Spain corresponds to dental treatment, a service that has very limited availability under the Spanish NHS. This finding highlights the need to formulate policies aimed at enhancing dental cover, in order to reduce inequalities in health care and, consequently, enhance the population’s quality of life and health status.

## Introduction

The Spanish National Health Service (NHS) was top ranked in the World Economic Forum’s 2019 index [1], based on indicators such as access to health care and indices of respiratory infections, neonatal disease and cancer treatments, among others. In another international classification, Spain was ranked twelfth in 2015 [2]. One outcome of this effective health system is that life expectancy in Spain is among the highest in the world, at 80.9 years for men and 86.2 years for women [3].

The main aims and criteria of the NHS are effectiveness, efficiency, satisfaction and fairness [4]. In 2019, Spain invested 75 billion euros (€1,593 per capita) in health care, equivalent to 6.0% of its GDP [5]. Comparable figures elsewhere include 9.9% of GDP invested by Germany (€4,108 per capita), 9.3% by France (€3,355 per capita) and 6.4% by Italy (€1,921 per capita) [6].

The Spanish NHS is well regarded among the population: according to a 2016 report, 63.7% of Spanish citizens believe the health system functions well or very well [7]. The European Commission reported that, over a broad portfolio of services, the NHS covered 71% of the cost of healthcare in 2015 (compared to the EU average of 79%). Direct payments by users accounted for a further 24%, compared to an EU average of 15% [8]. One of these direct payments corresponds to dental services, an essential provision which, nonetheless, has never been included in the coverage of the Spanish NHS beyond diagnosis and emergency extractions [9]. In consequence, this treatment is usually financed through out-of-pocket payments by the individual. From this, it must be concluded that dental treatment is not officially viewed as an important area of health care, but merely an aesthetic issue: indeed, in 2020, 27% of the Spanish population considered oral health to be a question of aesthetics [10]. Dental treatment for adults, therefore, is a major gap in NHS cover, together with ophthalmology for children and adults, and hearing aids for persons aged over 26 years [11].

In Spain, social class is a determining factor in the use of dental services, which are mainly accessed privately (only one in ten people use the public dental service, and mostly in childhood). It has been observed that people with a lower level of personal income have greater need of oral care and lower levels of dental restoration, and only seek treatment when more severe problems arise. Thus, in 2017 while 62.4% of the population with a higher level of income were attended by a dentist during the previous year, this value fell to 38.7% among those less well off. Similarly, 31% of the former group had postponed visiting the dentist for more than a year, versus 51.4% among those with less income [12]. Among households whose main breadwinner was classed as skilled or highly skilled, only 4% failed to obtain dental treatment due to financial problems, but among those where the breadwinner was less highly qualified, this value rose to 25% [13].

In greater detail, 14.08% of citizens lack the financial means to access the dental services they need [14]. A 2015 report found that for economic reasons 21% of survey respondents had not been to the dentist in the last two years. However, by 2020 this figure had fallen to 15% [10]. Moreover, 22% of the population had suffered tooth or gum pain in the last twelve months, and 19% had difficulty eating due to dental problems [10]. Regarding the treatment sought, 86% of those who visited a dentist did so at an independent private clinic, 11% attended one run by their insurance company [11] and only 3% were treated by a public-sector dentist [10]. By type of treatment, 30% of users visited the dentist for a check-up, 27% for a cleaning, 13% for a filling, 8% for pain relief, 6% for an implant, 6% for an extraction, 4% to remedy a fallen or broken tooth, 2% had gum problems, 2% presented misaligned teeth and 1% required root canal treatment or a crown [10].

A recent study found that on average 20% of Spanish households spent more than 10% of their income on health care, while 4.42% spent more than 40% of their income for this purpose, during the period 2008–2015 [15]. This study did not specify the type of health service or assistance referred to. A later study reported that 7.36% of households dedicated over 10% of their income to paying for dental care, and that 2.05% exceeded the 40% threshold for this purpose. These figures represent 36.32% and 51.34%, respectively, of out-of-pocket payments for health treatment [16].

Little international research has been conducted in this area, but one study reported that 16.5% of households in Saudi Arabia spend more than 10% of their income on dental health, while 5.5% spend over 40% of their income for this purpose [17]. Similarly, in Ukraine 6.8% of households spend more than 40% of their income on dental treatment. Another study has reported that in a range of low- and middle-income countries, 7% of households dedicate more than 40% of their income to dental treatment [18]. Furthermore, as GDP per capita increases, in countries with a solid Welfare State so does the percentage of resources that households allocate to payments for dental services, as does the proportion of adults who use these services [19]. However, there are important differences of public expenditure in the European Union: while Germany has the highest rate of public cover for dental health (meeting 68% of the cost), followed by Slovakia (53%), the average level in OECD countries is 29% and in Spain it is just 1% [20].

Other researchers have examined disparities in out-of-pocket expenditure, comparing various European countries (although not Spain), finding evidence that the lower a person’s level of education and income, the less likely they are to incur out-of-pocket expenses for dental services, either because these countries provide public-sector cover or because households with lower incomes are entitled to free care. However, as levels of education and income increase, so does personal expenditure on dental services [21]. The same study also concluded that in the USA dental payments by persons not entitled to free care are higher than elsewhere, and that these payments increase with income and level of education. They are also higher for persons with missing teeth, or who are in poor health, compared to those who have insurance cover, whose teeth are in generally good condition and whose general health status is good to excellent [21].

The question of inequality in access to health services has been analysed extensively, both in general [22] and with specific regard to dental services for children in Spain [23]. The latter study concluded that although children’s access to these services improved between 1987 and 2011, socioeconomic inequalities not only persisted but increased, and that children from households with a low socioeconomic level continued to make little use of dental services. For this reason, in the 1990s some regional administrations in Spain, including Navarre and the Basque Country, created free dental care programmes for children [24]. These programmes led to a 10–15% increase in the use made of dental services, compared to those regions that did not include these programmes [25]. In this respect, too, researchers have detected inequalities affecting dental access by the immigrant population, whose frequency of visits to the dentist is significantly lower than that of the native population [26].

In Spain, responsibilities for health services have been devolved to the regions, and so benefits, resources and facilities vary geographically [27]. In 2019, for example, investment in health care ranged from the 3.7% of GDP invested by the Madrid administration (€1,340 per capita), to 4.9% in Catalonia (€1,515 per capita), 7.6% in Murcia (€1,638 per capita) and 8.6% in Extremadura (€1,682 per capita) [5]. However, it has been suggested that the most important inequalities as concerns health are personal, not territorial, and are more strongly associated with the ‘intra’ than with the ‘inter’ management of resources [27].

The aim of the present study is to determine the incidence and concentration of catastrophic out-of-pocket spending on health and dental health services by households, according to the region of residence.

## Material and methods

Our analysis is based on microdata from the Spanish Family Budget Survey (FBS) [28, 29], which, for the three years considered, compiled socioeconomic and demographic data from 65,700 households (22,021 in 2008, 21,625 in 2011 and 22,054 in 2015). The information obtained includes data on the standard of living, income and professional activity of the reference person in the household, together with details of the nature and destination of annual household consumption expenses (equipment, housing, nutrition, health, education, tourism, etc.). These expenses are categorised according to the Classification of Individual Consumption by Purpose (COICOP), which contains the following modules: 1. Food and non-alcoholic drinks; 2. Alcoholic drinks, tobacco and narcotics; 3. Articles of clothing and footwear; 4. Housing, water, electricity, gas and other fuels; 5. Furniture, household equipment and current expenses for maintenance of the home; 6. Health; 7. Transportation; 8. Communications; 9. Leisure, shows and culture; 10. Teaching; 11. Hotels, cafes and restaurants; 12. Other goods and services.

Module number six refers to health spending, under the following headings: treatment devices and materials, medical services, dental services, clinical analysis services and X-ray centres, non-hospital auxiliary medical services, other out-of-hospital services, hospital services and non-disaggregated health expenses. The sum of these items, together with the subheading ‘dental services’, are the two out-of-pocket variables analysed in our study, i.e. household out-of-pocket spending on health care (OOP-H) and on dental care (OOP-D). This information is collected quarterly, via questionnaires, categorised by codes and then annualised by the Spanish Institute of Statistics for its final presentation.

We also considered household income, transformed into equivalent income. In line with previous work in this field [30, 31], the number of equivalent members of the household was calculated using the modified OECD scale, which assigns a value of 1 to the main breadwinner, 0.5 to each member of the household aged 14 years or over, and 0.3 to each member younger than 14 years [32]. Income per consumption unit or equivalent member was then obtained by dividing the total household income by the number of consumption units or equivalent members.

### Measuring catastrophic health expenditure

Using the methodology proposed by Wagstaff and van Doorslaer [33], we define a dummy variable E_i_ that takes the value 1 when the out-of-pocket spending (OOP-H and OOP-D) of household *i* as a proportion of its income (y_i_) exceeds the income threshold (z_cat_); in other words, (OPS_i_/y_i_) > z_cat_, and 0 otherwise. Household expenditure is considered catastrophic when the OOP-H / OOP-D exceeds the income threshold. The number of households in this situation, i.e. the proportion of households exposed to catastrophic health expenditure due to OOP-H / OOP-D (H_cat_), which quantifies the catastrophic incidence of OOP-H / OOP-D, is defined as:

1 $${H}_{cat}=\frac{1}{N}\sum_{i=1}^{n}{E}_{i}={\mu }_{E}$$

where N is the sample size and ${\mu }_{E}$ is the sample mean of E_i_.

Finally, we analyse the concentration of OOP-H / OOP-D in households with higher incomes and in those with lower incomes, by calculating the Concentration Index (CI). This index reveals the existence or absence of inequality in the distribution of individuals at risk of financial catastrophe, according to their socioeconomic status [34]. A CI close to zero means there is no socioeconomic inequality regarding the distribution of catastrophic OOP-H / OOP-D; a negative value indicates a high concentration of catastrophic payments in this respect among the poorest households; and a positive value suggests that households with higher incomes are exposed to the risk of catastrophic OOP-H / OOP-D [35]. The general formulation for the CI is:

2 $$CI=1-2{\int }_{0}^{1}L\left(p\right)dp$$

For the purposes of our study, the CI can be expressed more precisely as shown in Eq. 3, which shows that the value of the CI is equal to the covariance between the value of catastrophic OOP-H / OOP-D (y_i_) and the relative classification of individuals according to their income level (R_i_) divided by the average OOP-H / OOP-D (*μ*).

3 $$CI(y)=\frac{2}{\mu }cov({y}_{i},{R}_{i})$$

Potential CI values range from -1 to 1 (i.e., -1 ≤ CI ≤ 1, or *y*' – 1 ≤ CI ≤ 1 – *y*', where *y'* is the average of *y*, and *y* is the catastrophic OOP-H / OOP-D services.

Our study uses the income thresholds most commonly cited in the literature (z_cat_), i.e. 10%, 20%, 30% and 40%.

All statistical calculations were performed using Stata 16.0 (StataCorp LP, College Station, TX).

## Results

Tables 1, 2 and 3 summarise the sociodemographic information obtained for the study sample for the years 2008, 2011 and 2015, respectively. On average, in 2015, the family breadwinner was male (in 66.25% of households versus 72.66% in 2008 and 69.28% in 2011), aged 41 to 64 years (47.34% versus 44.74% in 2008 and 45.57% in 2011), married (54.02% in comparison with 60.62% in 2008 and 57.32% in 2011), had a medium level of education (48.82% versus 43.97% in 2008 and 50.16% in 2011), was in employment (56.78% respect to 62.28% in 2008 and 57.58% in 2011), had a low level of income (46.54% versus 41.24% in 2008 and 44.67% in 2011), and lived in a region with a high level of GDP per capita (39.86% in comparison to 39.96% in 2008 and 39.95% in 2011) and in an urban environment (85.63%, respect to 84.67% in 2008 and 85.09% in 2011).

**Table 1:** Descriptive statistics of the sociodemographic variables of households in Spain based on the percentage of resources they dedicate to dental care payments. Year 2008

	Total	Threshold < 10%	10% ≤ Threshold < 20%	20% ≤ Threshold < 30%	30% ≤ Threshold < 40%	Threshold ≥ 40%
Gender						
Male	72.66%	72.70%	76.64%	66.54%	63.61%	71.21%
Female	27.34%	27.30%	23.36%	33.46%	36.39%	28.79%
Age of head of household (years)						
Less than or equal to 40	27.05%	27.54%	19.32%	12.65%	12.78%	15.49%
41–64	44.74%	44.47%	52.12%	50.10%	45.34%	50.88%
More than or equal to 65	28.21%	27.99%	28.56%	37.25%	41.88%	33.63%
Marital Status						
Married	60.62%	60.45%	71.98%	60.52%	64.02%	53.92%
Single	19.61%	19.87%	13.22%	12.46%	12.52%	17.92%
Separated / Divorced	7.03%	6.94%	5.41%	11.30%	2.13%	15.61%
Widowed	12.74%	12.74%	9.39%	15.72%	21.33%	12.55%
Educational level						
Low level (Primary school incomplete, primary or equivalent)	30.21%	30.11%	31.37%	33.36%	34.13%	32.93%
Middle level (Secondary school / middle level professional)	43.97%	43.84%	46.35%	47.95%	44.93%	46.66%
University degree or equivalent (University degree or equivalent)	25.82%	26.05%	22.28%	18.69%	20.94%	20.41%
Activity Status						
Employed	62.28%	62.59%	59.07%	53.61%	44.79%	55.07%
Unemployed	4.73%	4.69%	7.93%	5.38%	2.54%	2.87%
Receiving earnings-related pension	28.04%	27.73%	29.67%	36.00%	46.22%	39.00%
Other situations (homecare, student, etc.)	4.95%	4.99%	3.33%	5.01%	6.45%	3.06%
Household Monthly Income						
Low level (less than €1,200)	41.24%	40.84%	45.93%	48.10%	45.72%	61.42%
Middle level (€1,200—€2,500)	42.42%	42.60%	40.60%	38.16%	44.87%	32.96%
High level (more than €2,500)	16.34%	16.56%	13.47%	13.74%	9.41%	5.62%
GDP per capita						
Low	31.02%	31.11%	31.22%	26.51%	30.57%	27.39%
Middle	29.02%	29.07%	27.86%	25.38%	19.83%	33.38%
High	39.96%	39.82%	40.92%	48.11%	49.60%	39.23%
Place of residence Urban (Ref. Rural)						
Rural	15.33%	15.37%	15.06%	16.56%	17.99%	9.36%
Urban	84.67%	84.63%	84.94%	83.44%	82.01%	90.64%
N	22,021	21,051	440	211	84	235

Threshold: refers to the relationship between the out-of-pocket payment (in health care and/or in dental care) and the level of equivalent income of the household

**Table 2:** Descriptive statistics of the sociodemographic variables of households in Spain based on the percentage of resources they dedicate to dental care payments. Year 2011

	Total	Threshold < 10%	10% ≤ Threshold < 20%	20% ≤ Threshold < 30%	30% ≤ Threshold < 40%	Threshold ≥ 40%
Gender						
Male	69.28%	68.96%	73.50%	71.55%	70.49%	76.38%
Female	30.72%	31.04%	26.50%	28.45%	29.51%	23.62%
Age of head of household (years)						
Less than or equal to 40	25.60%	26.34%	18.72%	18.30%	12.01%	9.50%
41–64	45.57%	44.89%	56.55%	50.45%	58.23%	52.84%
More than or equal to 65	28.83%	28.77%	24.73%	31.25%	29.76%	37.66%
Marital Status						
Married	57.32%	56.55%	67.99%	64.25%	64.22%	70.99%
Single	21.21%	21.67%	15.41%	20.67%	10.44%	12.23%
Separated / Divorced	12.50%	12.70%	8.53%	9.02%	16.42%	10.26%
Widowed	8.97%	9.08%	8.07%	6.06%	8.92%	6.52%
Educational level						
Low level (Primary school incomplete, primary or equivalent)	20.99%	21.15%	14.43%	20.50%	20.18%	25.09%
Middle level (Secondary school / middle level professional)	50.16%	49.86%	57.76%	49.15%	49.07%	53.23%
University degree or equivalent (University degree or equivalent)	28.85%	28.99%	27.81%	30.35%	30.75%	21.68%
Activity Status						
Employed	57.58%	57.61%	61.38%	58.27%	51.19%	50.63%
Unemployed	8.86%	8.85%	11.13%	5.03%	11.75%	7.11%
Receiving earnings-related pension	27.74%	27.58%	23.55%	33.04%	34.43%	37.60%
Other situations (homecare, student, etc.)	5.82%	5.96%	3.94%	3.66%	2.63%	4.66%
Household Monthly Income						
Low level (less than €1,200)	44.67%	44.79%	43.10%	46.96%	40.50%	41.14%
Middle level (€1,200—€2,500)	40.24%	39.99%	42.57%	43.39%	41.12%	46.68%
High level (more than €2,500)	15.09%	15.22%	14.33%	9.65%	18.38%	12.18%
GDP per capita						
Low	31.26%	31.33%	31.25%	33.14%	27.03%	27.57%
Middle	28.79%	28.85%	27.97%	29.07%	27.28%	27.80%
High	39.95%	39.82%	40.78%	37.79%	45.69%	44.63%
Place of residence Urban (Ref. Rural)						
Rural	14.91%	14.84%	12.85%	17.49%	20.49%	18.37%
Urban	85.09%	85.16%	87.15%	82.51%	79.51%	81.63%
N	21,625	20,056	710	275	165	419

Threshold: refers to the relationship between the out-of-pocket payment (in health care and/or in dental care) and the level of equivalent income of the household

**Table 3:** Descriptive statistics of the sociodemographic variables of households in Spain based on the percentage of resources they dedicate to dental care payments. Year 2015

	Total	Threshold < 10%	10% ≤ Threshold < 20%	20% ≤ Threshold < 30%	30% ≤ Threshold < 40%	Threshold ≥ 40%
Gender						
Male	66.25%	66.08%	68.24%	75.72%	73.37%	66.31%
Female	33.75%	33.92%	31.76%	24.28%	26.63%	33.69%
Age of head of household (years)						
Less than or equal to 40	21.37%	21.69%	20.22%	14.64%	6.86%	8.65%
41–64	47.34%	47.28%	49.07%	50.28%	57.57%	42.38%
More than or equal to 65	31.29%	31.03%	30.71%	35.08%	35.57%	48.97%
Marital Status						
Married	54.02%	53.81%	59.75%	65.55%	56.43%	50.77%
Single	22.85%	23.06%	22.05%	19.78%	11.18%	14.32%
Separated / Divorced	10.21%	10.19%	8.82%	8.52%	10.54%	16.07%
Widowed	12.92%	12.94%	9.38%	6.15%	21.85%	18.84%
Educational level						
Low level (Primary school incomplete, primary or equivalent)	19.80%	19.84%	14.88%	20.01%	23.66%	24.26%
Middle level (Secondary school / middle level professional)	48.82%	48.61%	52.58%	46.41%	51.58%	58.71%
University degree or equivalent (University degree or equivalent)	31.38%	31.55%	32.54%	33.58%	24.76%	17.03%
Activity Status						
Employed	56.78%	57.22%	56.17%	47.39%	43.87%	34.08%
Unemployed	8.24%	8.28%	6.89%	10.35%	1.58%	8.48%
Receiving earnings-related pension	29.22%	28.77%	31.72%	39.08%	46.64%	47.03%
Other situations (homecare, student, etc.)	5.76%	5.73%	5.22%	3.18%	7.91%	10.41%
Household Monthly Income						
Low level (less than €1,200)	46.54%	46.38%	46.82%	44.24%	44.19%	61.77%
Middle level (€1,200—€2,500)	39.98%	40.01%	39.24%	42.40%	48.17%	33.55%
High level (more than €2,500)	13.48%	13.61%	13.94%	13.36%	7.64%	4.68%
GDP per capita						
Low	31.54%	31.69%	29.27%	30.82%	23.95%	27.60%
Middle	28.60%	28.46%	31.56%	31.39%	40.58%	28.10%
High	39.86%	39.85%	39.17%	37.79%	35.47%	44.30%
Place of residence Urban (Ref. Rural)						
Rural	14.37%	14.41%	12.86%	17.51%	9.89%	13.17%
Urban	85.63%	85.59%	87.14%	82.49%	90.11%	86.83%
N	22,054	21,000	472	194	124	264

Threshold: refers to the relationship between the out-of-pocket payment (in health care and/or in dental care) and the level of equivalent income of the household

Tables 4, 5 and 6 show the data collected, by regions and the national total, for catastrophic OPS on dental care, at each of the thresholds considered, together with the incidence of health spending and its proportion of total spending in 2008, 2011 and 2015. These tables show that in 2008 Spanish households spent an annual average of €987.38 (SD: €2,756.05) on health services. Moreover, 11.12% of households dedicated 10% or more of their total income to health payments, while 4.73% spent 20% or more, 2.67% spent 30% or more and 1.79% spent 40% or more in this respect. With specific regard to catastrophic OOP-D, the results show for the same year Spanish households disbursed an annual average of €439.40 (SD: €2,222.13), while 4.19% spent 10% of their income (38.02% of the total catastrophic OOP-H), 2.27% spent 20% of income (47.70% of the total), 1.37% spent 30% of income (50.37% of the total) and 1.02% spent 40% of income (55.78% of the total).

**Table 4:** Prevalence, attributable percentage of total catastrophic health expenditure (CHE) and average annual spending (standard deviation) of financial catastrophism due to out-of-pocket payments for health, dental care, in Spain (€). Year 2008

Region (Nª household)	YEAR 2008	Threshold 10%	Threshold 20%	Threshold 30%	Threshold > or equal to 40%	Average cost per item (Standard deviation)
**Andalusia (n:2328)**	Catastrohic health expenditure	11,59%	4,51%	2,62%	1,67%	998,17 € (sd: 3.413,55)
	Catastrohic dental expenditure	4,81%	2,41%	1,50%	1,07%	471,49 € (sd: 3.180,32)
	Catas. dental/catas. health	41,48%	53,33%	57,38%	64,10%	
**Aragon (n:927)**	Catastrohic health expenditure	12,18%	4,63%	2,58%	1,72%	1070,82€(sd:2.520,42)
	Catastrohic dental expenditure	5,50%	2,48%	1,94%	1,40%	544,13€(sd:2.148,77)
	Catas. dental/catas. health	45,13%	53,49%	75,00%	81,25%	
**Asturias (n:816)**	Catastrohic health expenditure	10,78%	5,26%	3,06%	1,96%	1.089,75€(sd:3.334,69)
	Catastrohic dental expenditure	4,17%	2,94%	1,84%	1,35%	550,68€(sd:2.900,75)
	Catas. dental/catas. health	38,64%	55,81%	60,00%	68,75%	
**Balearic Islands (n:803)**	Catastrohic health expenditure	9,46%	3,73%	2,24%	1,24%	993,89€(sd:2.646,34)
	Catastrohic dental expenditure	3,24%	1,74%	0,87%	0,75%	426,81€(sd:2.057,05)
	Catas. dental/catas. health	34,21%	46,67%	38,89%	60,00%	
**Canary Islands (n:980)**	Catastrohic health expenditure	11,73%	4,38%	2,65%	1,32%	1.008,83€(sd:3.410,37)
	Catastrohic dental expenditure	3,78%	1,43%	0,82%	0,41%	373,61€(sd:3.108,10)
	Catas. dental/catas. health	32,17%	32,56%	30,77%	30,77%	
**Cantabria (n:749)**	Catastrohic health expenditure	11,08%	4,80%	2,80%	2,53%	978,34€(sd:2.429,34)
	Catastrohic dental expenditure	4,41%	2,27%	1,74%	1,60%	418,29€(sd:1.743,23)
	Catas. dental/catas. health	39,76%	47,22%	61,90%	63,16%	
**Castille Leon (n:1440)**	Catastrohic health expenditure	9,93%	4,09%	2,43%	2,01%	984,15€(sd:3.476,16)
	Catastrohic dental expenditure	4,17%	2,01%	1,18%	0,97%	433,75€(sd:2.582,69)
	Catas. dental/catas. health	41,96%	49,15%	48,57%	48,28%	
**Castille-La Mancha (n:1098)**	Catastrohic health expenditure	9,65%	4,82%	2,64%	1,82%	922,12€(sd:2.405,53)
	Catastrohic dental expenditure	3,92%	2,73%	1,18%	0,91%	421,76€(sd:1.880,50)
	Catas. dental/catas. health	40,57%	56,60%	44,83%	50,00%	
**Catalonia (n:2047)**	Catastrohic health expenditure	10,74%	4,59%	2,49%	1,46%	1.127,13€(sd:3.361,28)
	Catastrohic dental expenditure	4,89%	2,54%	1,22%	0,83%	483,77€(sd:2.511,71)
	Catas. dental/catas. health	45,45%	55,32%	49,02%	56,67%	
**Valencia Region (n:1644)**	Catastrohic health expenditure	12,77%	5,59%	3,40%	2,37%	1.205,81€(sd:3.3310,47)
	Catastrohic dental expenditure	5,17%	2,92%	1,76%	1,40%	557,91€(sd:2.810,34)
	Catas. dental/catas. health	40,48%	52,17%	51,79%	48,97%	
**Extremadura (n:946)**	Catastrohic health expenditure	13,53%	6,02%	3,06%	2,11%	798,78€(sd:1746,77)
	Catastrohic dental expenditure	3,38%	1,90%	1,06%	0,85%	259,98€(sd:1.121,61)
	Catas. dental/catas. health	25,00%	31,58%	34,48%	40,00%	
**Galicia (n:1367)**	Catastrohic health expenditure	11,45%	4,97%	2,99%	1,68%	1.002,10€(sd:2.505,80)
	Catastrohic dental expenditure	4,17%	2,41%	1,39%	1,02%	433,16€(sd:2.156,80)
	Catas. dental/catas. health	36,31%	48,53%	46,34%	60,87%	
**Madrid Region (n:1458)**	Catastrohic health expenditure	10,35%	5,07%	2,53%	1,57%	1.243,47€(sd:3.377,05)
	Catastrohic dental expenditure	4,53%	2,95%	1,51%	0,96%	564,72€(sd:2.344,44)
	Catas. dental/catas. health	43,71%	58,11%	59,46%	60,87%	
**Murcia Region (n:996)**	Catastrohic health expenditure	10,54%	3,51%	2,51%	1,60%	960,31€(sd:2.126,92)
	Catastrohic dental expenditure	3,01%	1,31%	1,00%	0,70%	321,01€(sd:1.188,21)
	Catas. dental/catas. health	28,57%	37,14%	40,00%	43,75%	
**Navarre Region (n:1433)**	Catastrohic health expenditure	10,46%	4,74%	2,86%	1,95%	1.306,46€(sd:4.395,38)
	Catastrohic dental expenditure	4,40%	2,51%	1,88%	1,47%	678,34€(sd:3.810,31)
	Catas. dental/catas. health	42,00%	52,94%	65,85%	75,00%	
**Basque Country (n:2057)**	Catastrohic health expenditure	9,57%	4,61%	3,11%	2,13%	1.249,80€(sd:4.039,71)
	Catastrohic dental expenditure	5,01%	2,97%	1,99%	1,46%	705,24€(sd:3.707,82)
	Catas. dental/catas. health	52,28%	64,21%	64,06%	68,18%	
**Rioja (n:703)**	Catastrohic health expenditure	12,09%	5,83%	2,84%	2,13%	971,54€(sd:2.163,48)
	Catastrohic dental expenditure	4,69%	2,42%	1,28%	0,71%	410,29€(sd:1.700,53)
	Catas. dental/catas. health	38,82%	41,46%	45,00%	33,33%	
**Ceuta and Melilla (n:228)**	Catastrohic health expenditure	12,28%	3,94%	1,31%	0,87%	848,79€(sd:1.701,79)
	Catastrohic dental expenditure	2,19%	0,88%	0,44%	0,44%	293,63€(sd:1.267,41)
	Catas. dental/catas. health	17,86%	22,22%	33,33%	50,00%	
**National average**	Catastrohic health expenditure	11,12%	4,73%	2,67%	1,79%	987,38€(sd:2.756,05)
	Catastrohic dental expenditure	4,19%	2,27%	1,37%	1,02%	293,63€(sd:1.267,41)
	Catas. dental/catas. health	38,02%	47,70%	50,37%	55,78%

**Table 5:** Prevalence, attributable percentage of total catastrophic health expenditure (CHE) and average annual spending (standard deviation) of financial catastrophism due to out-of-pocket payments for health, dental care, in Spain (€). Year 2011

Region (Nª household)	YEAR 2008	Threshold 10%	Threshold 20%	Threshold 30%	Threshold > or equal to 40%	Average cost per item (Standard deviation)
**Andalusia (n:2396)**	Catastrohic health expenditure	10,02%	3,55%	2,00%	1,42%	804,78€(sd:2.001,60)
	Catastrohic dental expenditure	3,79%	1,79%	1,00%	0,79%	324,80€(sd:1.456,09)
	Catas. dental/catas. health	37,91%	50,58%	50,00%	55,88%	
**Aragon (n:955)**	Catastrohic health expenditure	7,64%	3,66%	2,09%	1,47%	812,47€(sd:2.383,44)
	Catastrohic dental expenditure	2,93%	1,78%	1,15%	0,83%	333,53€(sd:1.719,39)
	Catas. dental/catas. health	38,35%	48,57%	55,00%	57,14%	
**Asturias (n:821)**	Catastrohic health expenditure	7,19%	2,68%	1,46%	0,73%	781,20€(sd:2.401,58)
	Catastrohic dental expenditure	3,65%	1,70%	1,09%	0,48%	391,98€(sd:2.040,08)
	Catas. dental/catas. health	50,84%	63,63%	75,00%	66,66%	
**Balearic Islands (n:811)**	Catastrohic health expenditure	8,63%	3,08%	1,48%	0,62%	937,54€(sd:2.310,31)
	Catastrohic dental expenditure	2,95%	0,98%	0,86%	0,24%	377,98€(sd:1.770,63)
	Catas. dental/catas. health	34,28%	32,00%	58,33%	40,00%	
**Canary Islands (n:993)**	Catastrohic health expenditure	10,67%	3,93%	2,32%	1,41%	909,58€(sd:2.070,39)
	Catastrohic dental expenditure	3,22%	1,71%	0,90%	0,60%	324,77€(sd:1.429,56)
	Catas. dental/catas. health	30,18%	43,58%	39,13%	42,85%	
**Cantabria (n:762)**	Catastrohic health expenditure	11,68%	6,69%	3,67%	1,97%	1.030,42€(sd:2.519,20)
	Catastrohic dental expenditure	5,77%	3,93%	2,09%	1,31%	568,65€(sd:2.314,97)
	Catas. dental/catas. health	49,43%	58,82%	57,14%	66,66%	
**Castille Leon (n:1416)**	Catastrohic health expenditure	10,10%	4,17%	2,05%	1,62%	883,61€(sd:3.202,74)
	Catastrohic dental expenditure	4,37%	1,83%	1,20%	0,91%	440,48€(sd:2.885,55)
	Catas. dental/catas. health	43,35%	44,06%	58,62%	56,52%	
**Castille-La Mancha (n:1175)**	Catastrohic health expenditure	11,57%	5,96%	3,15%	1,96%	1,016,52€(sd:4.234,24)
	Catastrohic dental expenditure	5,02%	2,63%	1,70%	1,19%	552,99€(sd:3.866,28)
	Catas. dental/catas. health	43,38%	44,28%	54,05%	60,86%	
**Catalonia (n:1966)**	Catastrohic health expenditure	11,24%	4,73%	3,05%	2,03%	1.096,13€(sd:2.524,27)
	Catastrohic dental expenditure	4,83%	2,33%	1,37%	0,81%	485,86€(sd:1.881,79)
	Catas. dental/catas. health	42,98%	49,46%	45,00%	40,00%	
**Valencia Region (n:1724)**	Catastrohic health expenditure	10,50%	4,29%	2,44%	1,74%	905,10€(sd:2.726,68)
	Catastrohic dental expenditure	4,06%	2,20%	1,50%	1,10%	402,40€(sd:1.997,94)
	Catas. dental/catas. health	38,67%	51,35%	61,90%	63,33%	
**Extremadura (n:970)**	Catastrohic health expenditure	11,34%	4,12%	2,27%	1,03%	721,32€(sd:1.856,12)
	Catastrohic dental expenditure	3,29%	1,44%	1,13%	0,82%	257,04€(sd:1.429,93)
	Catas. dental/catas. health	29,09%	35,00%	50,00%	80,00%	
**Galicia (n:1321)**	Catastrohic health expenditure	11,43%	4,39%	2,42%	1,29%	991,30€(sd:2.568,14)
	Catastrohic dental expenditure	5,22%	2,64%	1,43%	0,75%	479,73€(sd:2.198,52)
	Catas. dental/catas. health	45,69%	60,34%	59,37%	58,82%	
**Madrid Region (n:1563)**	Catastrohic health expenditure	9,02%	3,71%	1,98%	1,34%	1.124,76€(sd:3.136,69)
	Catastrohic dental expenditure	4,60%	2,30%	1,34%	1,02%	570,33€(sd:2.723,64)
	Catas. dental/catas. health	51,06%	62,06%	67,74%	76,19%	
**Murcia Region (n:913)**	Catastrohic health expenditure	9,97%	3,83%	1,97%	1,20%	834,53€(sd:2.385,37)
	Catastrohic dental expenditure	3,61%	1,75%	0,76%	0,32%	268,78€(sd:1.038,86)
	Catas. dental/catas. health	36,26%	45,71%	38,88%	27,27%	
**Navarre Region (n:741)**	Catastrohic health expenditure	10,12%	3,78%	2,02%	1,75%	1.028,88€(sd:2.705,81)
	Catastrohic dental expenditure	4,58%	2,29%	1,48%	1,21%	491,93€(sd:2.382,64)
	Catas. dental/catas. health	45,33%	60,71%	73,33%	69,23%	
**Basque Country (n:2134)**	Catastrohic health expenditure	8,95%	4,31%	2,95%	2,11%	1.118,40€(sd:3.812,86)
	Catastrohic dental expenditure	4,26%	2,38%	1,64%	1,21%	540,12€(sd:2.959,51)
	Catas. dental/catas. health	47,64%	55,43%	55,55%	57,77%	
**Rioja (n:717)**	Catastrohic health expenditure	11,02%	4,60%	2,65%	1,95%	1.013,22€(sd:2.654,44)
	Catastrohic dental expenditure	4,18%	2,51%	1,39%	1,11%	484,37€(sd:2.359,17)
	Catas. dental/catas. health	37,97%	54,54%	52,63%	57,14%	
**Ceuta and Melilla (n:247)**	Catastrohic health expenditure	11,34%	5,26%	2,43%	2,43%	943,71€(sd:1.925,72)
	Catastrohic dental expenditure	4,04%	2,42%	1,61%	1,61%	293,64€(sd:1.131,92)
	Catas. dental/catas. health	35,71%	46,15%	66,66%	66,66%	
**National Average**	Catastrohic health expenditure	10,14%	4,26%	2,36%	1,56%	892,29€(sd:2.495,77)
	Catastrohic dental expenditure	4,13%	2,15%	1,31%	0,91%	399,44€(sd:1.978,24)
	Catas. dental/catas. health	41,01%	50,35%	56,57%	57,94%

**Table 6:** Prevalence, attributable percentage of total catastrophic health expenditure (CHE) and average annual spending (standard deviation) of financial catastrophism due to out-of-pocket payments for health, dental care, in Spain (€). Year 2015

Region (Nª household)	YEAR 2008	Threshold 10%	Threshold 20%	Threshold 30%	Threshold > or equal to 40%	Average cost per item (Standard deviation)
**Andalusia (n:2401)**	Catastrohic health expenditure	21,74%	11,41%	6,91%	4,91%	896,06€(sd:2.251,33)
	Catastrohic dental expenditure	8,62%	4,75%	3,17%	2,21%	393,67€(sd:1.952,55)
	Catas. dental/catas. health	39,66%	41,61%	45,78%	44,92%	
**Aragon (n:984)**	Catastrohic health expenditure	18,19%	8,94%	5,89%	4,27%	1.064,03€(sd:2.961,16)
	Catastrohic dental expenditure	7,83%	4,57%	3,46%	3,05%	524,88€(sd:2.500,09)
	Catas. dental/catas. health	43,02%	51,14%	58,62%	71,43%	
**Asturias (n:886)**	Catastrohic health expenditure	15,91%	6,77%	4,85%	3,61%	1.057,39€(sd:2.872,95)
	Catastrohic dental expenditure	5,64%	2,82%	2,26%	1,81%	386,68€(sd:1.699,32)
	Catas. dental/catas. health	35,46%	41,67%	46,51%	50,00%	
**Balearic Islands (n:774)**	Catastrohic health expenditure	20,54%	10,72%	6,72%	4,52%	1.199,61€(sd:3.414,05)
	Catastrohic dental expenditure	10,34%	5,43%	4,13%	2,84%	662,47€(sd:3.092,21)
	Catas. dental/catas. health	50,31%	50,60%	61,54%	62,86%	
**Canary Islands (n:1013)**	Catastrohic health expenditure	16,58%	9,67%	5,43%	3,75%	962,68€(sd:2.050,06)
	Catastrohic dental expenditure	3.95%	2,47%	1,58%	1,09%	296,93€(sd:1.368,66)
	Catas. dental/catas. health	23,81%	25,51%	29,09%	28,95%	
**Cantabria (n:755)**	Catastrohic health expenditure	17,48%	9,14%	5,17%	3,71%	996,78€(sd:2.602,78)
	Catastrohic dental expenditure	7,15%	4,50%	3,31%	2,65%	482,64€(sd:2.183,18)
	Catas. dental/catas. health	40,91%	49,28%	64,10%	71,43%	
**Castille Leon (n:1477)**	Catastrohic health expenditure	9,48%	3,79%	2,37%	1,83%	850,39€(sd:2.242,03)
	Catastrohic dental expenditure	2,84%	1,08%	0,88%	0,68%	382,43€(sd:1.786,79)
	Catas. dental/catas. health	30,00%	28,57%	37,14%	37,04%	
**Castille-La Mancha (n:1217)**	Catastrohic health expenditure	14,05%	7,07%	4,60%	3,20%	1.067,16€(sd:3.051,22)
	Catastrohic dental expenditure	5,01%	3,20%	2,63%	2,05%	558,45€(sd:2.699,03)
	Catas. dental/catas. health	35,67%	45,35%	57,14%	64,10%	
**Catalonia (n:2022)**	Catastrohic health expenditure	7,96%	4,15%	2,27%	1,53%	1.171,42€(sd:3.574,55)
	Catastrohic dental expenditure	2,52%	1,48%	0,99%	0,64%	592,05€(sd:3.195,78)
	Catas. dental/catas. health	31,68%	35,71%	43,48%	41,94%	
**Valencia Region (n:1707)**	Catastrohic health expenditure	10,66%	4,80%	2,93%	2,11%	1.112,98€(sd:2.561,02)
	Catastrohic dental expenditure	3,93%	2,40%	1,70%	1,29%	458,36€(sd:1.899,14)
	Catas. dental/catas. health	36,81%	50,00%	58,00%	61,11%	
**Extremadura (n:995)**	Catastrohic health expenditure	16,58%	8,34%	5,83%	4,12%	905,01€(sd:2.260,85)
	Catastrohic dental expenditure	5,13%	3,22%	2,51%	1,81%	380,27€(sd:1.991,19)
	Catas. dental/catas. health	30,91%	38,55%	43,10%	43,90%	
**Galicia (n:1346)**	Catastrohic health expenditure	12,56%	6,76%	4,53%	2,82%	1.195,36€(sd:3.264,32)
	Catastrohic dental expenditure	4,90%	2,82%	1,78%	1,34%	456,01€(sd:2.099,13)
	Catas. dental/catas. health	39,05%	41,76%	39,34%	47,37%	
**Madrid Region (n:1640)**	Catastrohic health expenditure	9,51%	3,90%	1,95%	1,16%	1.160,80€(sd:2.380,07)
	Catastrohic dental expenditure	4,39%	2,01%	1,28%	0,98%	528,71€(sd:2.025,39)
	Catas. dental/catas. health	46,15%	51,56%	65,63%	84,21%	
**Murcia Region (n:904)**	Catastrohic health expenditure	21,13%	9,62%	4,87%	2,99%	933,43€(sd:2.083,80)
	Catastrohic dental expenditure	6,42%	3,21%	1,77%	1,00%	372,60€(sd:1.693,20)
	Catas. dental/catas. health	30,37%	33,33%	36,36%	33,33%	
**Navarre Region (n:739)**	Catastrohic health expenditure	21,24%	9,88%	5,55%	3,38%	1.183,11€(sd:2.792,74)
	Catastrohic dental expenditure	7,85%	3,65%	2,84%	2,44%	508,82€(sd:2.130,34)
	Catas. dental/catas. health	36,94%	36,99%	51,22%	72,00%	
**Basque Country (n:2219)**	Catastrohic health expenditure	5,68%	2,75%	1,71%	1,22%	1.212,66€(sd:3.869,82)
	Catastrohic dental expenditure	2,57%	1,44%	1,17%	0,72%	531,55€(sd:3.080,38)
	Catas. dental/catas. health	45,24%	52,46%	68,42%	59,26%	
**Rioja (n:729)**	Catastrohic health expenditure	19,20%	9,05%	5,35%	3,84%	947,19€(sd:2.298,04)
	Catastrohic dental expenditure	7,54%	4,12%	3,02%	2,47%	452,67€(sd:1.918,27)
	Catas. dental/catas. health	39,29%	45,45%	56,41%	64,29%	
**Ceuta and Melilla (n:245)**	Catastrohic health expenditure	28,16%	19,18%	10,20%	8,57%	1.475,83€(sd:5.276,07)
	Catastrohic dental expenditure	8,57%	6,12%	3,27%	3,27%	757,05€(sd:5.082,93)
	Catas. dental/catas. health	30,43%	31,91%	32,00%	38,10%	
**National Average**	Catastrohic health expenditure	15,93%	8,11%	4,84%	3,42%	1.020.63€(sd:2.726,68)
	Catastrohic dental expenditure	5,96%	3,29%	2,32%	1,80%	459,28€(sd:2.231,45)
	Catas. dental/catas. health	36,98%	41,75%	49,66%	54,24%	

Over the 2008—2015 data series, there is a certain stability in the values obtained, with a slight decrease between 2008 and 2011 and then a small rebound in 2015, in the percentages of incidence, both for overall health care spending and for dental services. The proportion of dental health spending within overall catastrophic OOP-H remained stable for all three years considered. However, although the incidence increased from 2011 to 2015, the proportion of catastrophic OOP-D compared to total spending decreased, for all thresholds.

Tables 4, 5 and 6 also present the regional variations observed for the same variables, which reflect a certain degree of homogeneity. For example, in 2008, Aragón reported the highest values, with 5.50% of households exposed to catastrophic OOP-D at the 10% threshold; by contrast, in the Basque Country 2.97% of households dedicated 20% or more of their income on dental health payments, and 1.99% spent 30% or more in this respect. In Cantabria 1.60% of households spent 40% or more of their income on access to these services. The lowest values were recorded for Ceuta and Melilla, with 2.19% of households exposed to catastrophic OOP-D at the 10% threshold, and Murcia, where the incidence was 3.01%. For the highest threshold, the Canary Islands reported the lowest percentage, i.e., that 0.41% of households dedicated 40% or more of their income for OOP-D. The values obtained for 2011 and 2015, although varying among the regions, were fundamentally stable as concerns the rates of catastrophic out-of-pocket spending, both for dental health in absolute terms and as a proportion of catastrophic OOP-H in general.

Table 7 shows the value of out-of-pocket concentration indices for overall health and for dental health, by regions, and the national average for 2008, 2011 and 2015. For 2008, the national average CI was -0.14, which indicates significant concentration of the payment burden within lower-income households; by 2011, the CI had decreased to -0.11, in 2015 it has a similar value, -0.11 (*p* < 0.000 in every case). The CI values for OOP-D were -0.13, -0.06 and -0.10 for 2008, 2011 and 2015, respectively, which is in line with the payments for health services in general. The distribution of this spending presented a certain concentration within low-income households, and by regions varied from -0.26 in Murcia to 0.02 in Andalusia, in 2015.

**Table 7:** Concentration index of out-of-pocket payments for health and dental health services by region in Spain. Years 2008, 2011 and 2015

	**2008**	**2011**	**2015**	**2008**	**2011**	**2015**
	**OOP health services**			**OOP dental health services**		
**Andalusia**	-0,13***	-0,12***	-0,07***	-0,15**	-0,15**	0,019
**Aragon**	-0,06	-0,07	-0,15**	-0,03	-0,05	-0,23**
**Asturias**	-0,05	-0,01	-0,09	-0,02	0,04	-0,14
**Balearic Islands**	-0,14**	0,00	-0,03	-0,02	-0,00	-0,02
**Canary Islands**	-0,04	-0,11**	-0,06***	0,11	-0,11	-0,02
**Cantabria**	-0,22***	-0,11**	0,01	-0,17*	-0,04	0,02
**Castille Leon**	-0,20***	-0,06	-0,15***	-0,10	0,08	-0,17**
**Castille-La Mancha**	0,02	-0,14**	-0,10**	0,12	-0,12	-0,13
**Catalonia**	-0,12***	-0,10***	-0,18***	-0,14**	-0,00	-0,20**
**Valencia Region**	-0,16***	-0,13***	-0,15***	-0,15**	-0,12	-0,13**
**Extremadura**	-0,21***	-0,13***	-0,10**	-0,18**	-0,09	-0,07
**Galicia**	-0,15***	-0,05	-0,12***	-0,17**	0,02	-0,15**
**Madrid Region**	-0,22***	-0,10**	-0,12***	-0,17**	-0,08	-0,02
**Murcia Region**	-0,17***	-0,03	-0,17***	-0,19**	0,02	-0,26**
**Navarre Region**	-0,10*	-0,10*	-0,08	-0,06	-0,06	-0,07
**Basque Country**	-0,23***	-0,28***	-0,08**	-0,25***	-0,16**	-0,04
**Rioja**	-0,16***	-0,13**	-0,11*	-0,19**	-0,02	-0,06
**Ceuta and Melilla**	-0,17	-0,14	-0,29	0,04	-0,28	-0,16
**National Average**	-0,14***	-0,11***	-0,12***	-0,13***	-0,06***	-0,10***

*OOP* Out of pocket payments

^*^Denotes significance at the level of *p*<0.10

^**^Denotes significance at the level of *p*<0.05

^***^Denotes significance at the level of *p*<0.01

## Discussion

The public-sector provision of dental care remains open to improvement, in view of the significant level of catastrophic out-of-pocket expenditure for this purpose, representing almost 50% of all such payments for health services, at all thresholds and in all regions. This finding is in line with the literature, according to which the two main causes of catastrophic out-of-pocket expenditure in Spain have traditionally been dental care and the purchase of medical devices, a pattern that is constant within all consumption quintiles [11]. The immediate consequence of this situation is a reduced frequency of visits to the dentist, which is less than half that observed in countries with more comprehensive public-sector cover (such as Germany and Denmark). In Spain, moreover, there is greater inequality in the pattern of access to dental services than is the case for other medical specialities [36]. The study data also reveal that although some regions include dental care in their supplementary service portfolio (for example, for children and for adults with dependency), this provision does not achieve significant differences in levels of catastrophic out-of-pocket spending. This lack of impact suggests that although these programmes may alleviate the financial burden on the population groups addressed, they probably do not achieve the desired effect among the population as a whole.

There is considerable inequality in the financing of dental services according to the public or private nature of the resources deployed. For example, in 2015, direct payments by households amounted to 3,962 million euros, while 49 million euros were spent by private companies, 62 million euros by the public sector and 19.3 million euros by mutual societies [37].

Significant differences were observed between the regions with the lowest and highest levels of average dental health expenditure per capita (Extremadura, with 35.45 euros, and Cantabria, with 87.22 euros, respectively). Among the highest-spending regions, Cantabria is followed by Castille Leon and Navarre Region, with 81.2 euros and 79.99 euros, respectively. Among those spending least, Extremadura is followed, at a significant distance, by the Canary Isles (52.07 euros) and Catalonia (55.23 euros). The national average is 65.33 euros [37].

The unequal distribution of financial burdens in the field of dental health care and the need to improve the fairness and effectiveness of health systems are important issues in Spain and elsewhere [38]. For example, a study conducted in Iran reported an uneven uptake of dental services; households with lower incomes made much less use of these services than those with higher incomes, for similar needs [39]. A similar pattern has been observed in European countries; thus, Poland has the highest concentration index among lower-income households, while Germany records the lowest level of inequality in OOP-D [40]. In the USA, an expansion of dental insurance cover would narrow the current gap in the use of these services; at present, there is significantly greater demand for dental care from citizens who have insurance cover in this respect than from those who do not [41].

In Spain, although the mean probability of never having visited a dentist fell from 49.5% in 1987 to 8.4% in 2011 [24], the health service continues to present room for improvement in various areas, chief among which is that of dental health [42]. Thus, more should be done to alleviate the inequalities observed in access to dental care, regarding not only preventive treatment, but also basic restoration and aesthetic services, which are currently only accessible to those with greater financial resources [9]. In this regard, it has been observed that in developed countries dental appearance can play a significant role in facilitating integration into society and in determining social position [43].

In this line, access to dental health services remains heavily dependent on individual circumstances, and is sometimes impossible in Spain. As a result, there is significant socioeconomic inequality and unmet needs in the field of dental care [11]. For this reason, fresh policies are needed to strengthen financial protection for households that are especially vulnerable to catastrophic OOP-H, with particular emphasis on dental care, and expanding the portfolio of oral care services available to them [11].

Apart from the aesthetic value of dental health care, deficiencies in this respect are associated with other pathologies, increasing the pressure on health system resources. In this respect, a recent study examined the association between periodontitis and the impact of SARS-Cov2 [44], finding that admission to an ICU due to COVID-19 infection was 3.5 times more likely for patients with advanced gum disease. Similarly, the need for assisted breathing was 4.6 times more likely, and the probability of death from COVID-19 was 8.8 times higher among these patients.

In addition, numerous studies have identified an association between untreated oral pathologies and the complications arising from cardiovascular disease [45] or chronic conditions such as diabetes [46], which increase their morbidity and mortality. Indeed, there is a general consensus that dental care should be more comprehensively addressed, as a priority for public health, via increased investment and focused public policies. Finally, measures should be taken to reduce the consumption of sugar, tobacco and alcohol [47].

Our study presents certain limitations, chiefly the fact that the households included in our study samples varied from one survey year to the next. If the data collection design used for each survey took this question into account and, moreover, provided more detailed information about respondents’ health, this would provide more study variables and enable us to optimise the statistical analysis, thus facilitating and strengthening the drawing of conclusions. A second limitation is that the data is somewhat older.

## Conclusions

To our knowledge, this is the first study to be conducted on catastrophic OPS on general and dental health services in Spain, disaggregated by regions.

In 2022, the Spanish Ministry of Health announced a proposal to expand the coverage of the common portfolio of the Spanish NHS services with respect to dental care services, with special attention to the following groups: children and adolescents, pregnant women, people with disabilities, the elderly and vulnerable populations. The main objective of this reform is to increase the coverage of healthcare benefits, through better intersectoral coordination, transversality and the introduction of new technologies. The cost of this proposal is expected to be 45.6 million euros [37].

According to the proposal, new policies in this area should be applied in two ways. On the one hand, as most dental spending concerns restorative work, more effort is needed on policies to promote oral health and to prevent disease, especially in the early stages of life (childhood and youth stages), fostering not only healthy nutrition, but also good habits of oral health. Appropriate interventions in primary and secondary schools such as awareness campaigns, the public provision (or subsidy) of dental care resources (toothbrushes, toothpaste, fluoridisation, etc.) and the design of health-focused diets would enhance nutritional education and help prevent dental diseases, both direct and indirect. In addition, public policies should focus oral health reviews on people in situations of financial vulnerability, who are otherwise less likely to seek dental treatment [12].

Our study identifies several interesting lines for future research, such as quantifying the level of out-of-pocket spending, per household and by regions, on dental care, and estimating the total cost of dental care needs that are currently not covered by the public health system. It would also be useful to determine the secondary health care needs provoked by prior dental pathologies, requiring subsequent and often costly medical attention. Finally, an analysis of the available data should be performed disaggregating by relevant sociodemographic variables, such as sex, education, financial status and place of residence, in order to generate profiles of access to and use of dental services, and the consequences of variations in this respect.

## Data Availability

The datasets generated and/or analysed during the current study are available from the INE (National Institute of Statistics), at: https://www.ine.es/dyngs/INEbase/es/operacion.htm?c=Estadistica_C&cid=1254736176806&menu=resultados&idp=1254735976608#!tabs-1254736194790
